# The Importance of Cognitive Appraisal and Social Support in Pregnancy During COVID-19 from an Interdisciplinary View

**DOI:** 10.1192/j.eurpsy.2022.76

**Published:** 2022-09-01

**Authors:** M. Howard

**Affiliations:** 1 Brown University, Warren Alpert Medical School, Providence, United States of America; 2 Women & Infants Hospital, Women’s Behavioral Health, Providence, United States of America

**Keywords:** Partial Hospital, Virtual Treatment, Postnatal Depression, Mother-Baby Unit

## Abstract

Aim: To describe how a US-based psychiatric Mother-Baby Unit adopted a virtual platform during the pandemic. Objectives: When the Covid-19 pandemic descended, mental health clinicians world-wide were faced with maintaining access and care delivery. Pregnant and newly postpartum women experienced the pandemic and lockdown through the lens of impending parenthood and new parenthood--amplifying distress, isolation, and lack of social and family support. The lockdown prevented those with most acute illness from accessing daily treatment in our structured, supportive intensive treatment setting. We acknowledged the urgency of developing a method to continue to treat our patients in a group environment that offered psychotherapy, psychopharmacology, dyadic and family intervention, and social support. Methods: The hospital purchased “Zoom for Health” platform to ensure compliance with regulatory guidelines. Cameras for individual computers were purchased with philanthropy funds, obtaining them quickly, compared to waiting for hospital funding. The clinical team designed a schedule of groups and individual sessions, each with their own zoom link. Each morning, a team member, sent the daily schedule through the patient portal of the EMR. Conclusion: Before the pandemic, the census was 12 pts per day. The full-day program paused for 7-10 days although individual sessions and medication management were conducted by phone as the virtual platform was constructed. Once established, census resumed normal levels. Challenges to running the Day Hospital virtually included: technology glitches, family demands and distractions, and privacy concerns. Overall, the program was a success wherein women requiring intensive treatment were able to receive treatment and social support.

**Disclosure:**

No significant relationships.

